# ASSOCIATION OF BPSD AND CARE PARTNER OUTCOMES MEDIATED BY CARE PARTNER DISTRESS

**DOI:** 10.1093/geroni/igae098.1364

**Published:** 2024-12-31

**Authors:** Diane Berish

**Affiliations:** The Pennsylvania State University, University Park, Pennsylvania, United States

## Abstract

Behavioral and psychological symptoms of dementia (BPSD) include a range of symptoms that commonly occur during hospitalization and post-discharge. BPSD can be difficult to anticipate and can create challenges for family care partners which may lead to adverse care partner outcomes. Repeated measures data from the Fam-FFC study provides opportunity to examine the time-dependent relationship among BPSD, associated care partner distress, and care partner outcomes. Mediation models were used with Neuropsychiatric Inventory Questionnaire (NPI) scores at discharge from an acute care hospitalization, NPI scores of associated care partner distress at 2-months post discharge, and care partners outcomes at 6-months, including burden, strain, desire to institutionalize and level of preparedness for caregiving. Patient and care partner characteristics were included as covariates. Results showed that NPI severity had direct effects on desire to institutionalize (estimate=0.0754, p=0.0119) with no significant indirect effects. Care partner burden, NPI severity had statistically significant direct effects (estimate=0.3023, p=0.0038), with 53.4% (p<0.0001) mediational effect of associated care partner distress. Similarly, for care partner strain, NPI severity had statistically significant direct effects (estimate=0.21, p=0.003), with 48.8% (p<0.0001) mediational effect of associated care partner distress. There were no significant effects for the outcome of care partner preparedness. The repeated measures design study allows for the examination of these relationships over time and helps to build evidence for a mechanistic model. These results show the importance of assessment of BPSD presentation, particularly post-acute hospitalization, and providing support for care partners to reduce negative outcomes.

